# Effect of dexmedetomidine on postoperative systemic inflammation and recovery in patients undergoing digest tract cancer surgery: A meta-analysis of randomized controlled trials

**DOI:** 10.3389/fonc.2022.970557

**Published:** 2022-09-14

**Authors:** Wenjie Xu, Yuxiang Zheng, Zizheng Suo, Kailun Fei, Yalong Wang, Chao Liu, Shuai Li, Mingzhu Zhang, Yefan Zhang, Zhaoxu Zheng, Cheng Ni, Hui Zheng

**Affiliations:** ^1^ Department of Anesthesiology, National Cancer Center/National Clinical Research Center for Cancer/Cancer Hospital, Chinese Academy of Medical Sciences and Peking Union Medical College, Beijing, China; ^2^ Department of Medical Oncology, National Cancer Center/National Clinical Research Center for Cancer/Cancer Hospital, Chinese Academy of Medical Sciences and Peking Union Medical College, Beijing, China; ^3^ Department of Hepatobiliary Surgery, National Cancer Center/National Clinical Research Center for Cancer/Cancer Hospital, Chinese Academy of Medical Sciences and Peking Union Medical College, Beijing, China; ^4^ Department of Colorectal Surgery, National Cancer Center/National Clinical Research Center for Cancer/Cancer Hospital, Chinese Academy of Medical Sciences and Peking Union Medical College, Beijing, China

**Keywords:** dexmedetomidine, immune function, postoperative cognitive dysfunction, digestive tract cancer, prognosis, meta-analysis

## Abstract

Perioperative immune function, postoperative cognitive function and prognosis are momentous issues for patients undergoing digestive tract cancer surgery. Studies have investigated the efficacy of dexmedetomidine (DEX) administration on these issues, but the results are inconsistent. Therefore, this meta-analysis aimed to summarize all the existing evidence and draw a conclusion more accurately on these associations. Trials were located through electronic searches of the PubMed, Embase, the Cochrane Library and Web of Science databases sources (from the establishment date of databases to April 2022). Bibliographies of the retrieved articles were checked. A total of 17 RCTs involving 1619 patients were included. The results showed that DEX decreased the level of C-reactive protein (SMD = -4.26, 95%CI: -6.16, -2.36), TNF-α (SMD = -4.22, 95%CI: -5.91, -2.54) and IL-6 (SMD = -2.71, 95%CI: -4.46, -0.97), and increased the level of IL-10 (SMD = 1.74, 95%CI: 0.25, 3.24). DEX also increased CD4+ T cells (SMD = 0.55, 95%CI: 0.29, 0.82) and CD4+/CD8+ ratio (SMD = 0.62, 95%CI: 0.24, 1.01). Thus, DEX was associated with alleviation of postoperative systemic inflammatory response and immune dysfunction. Furthermore, DEX increased mini-mental state examination scores at 12h (SMD = 1.10, 95%CI: 0.74,1.45), 24h (SMD = 0.85, 95%CI: 0.59, 1.11), 48h (SMD = 0.89, 95%CI: 0.50, 1.28) and 72h (SMD = 0.75, 95%CI: 0.38, 1.11) after surgery. DEX decreased the occurrence of postoperative cognitive dysfunction (POCD) at 24h (OR = 0.22, 95%CI: 0.11, 0.46) and 72h (OR = 0.39, 95%CI: 0.22, 0.68) after surgery. DEX decreased first flatus time (SMD = -1.55, 95%CI: -2.82, -0.27) and hospital stay (SMD = -1.23, 95%CI: -1.88, -0.59). Therefore, based on perioperative immune dysfunction alleviation, DEX attenuated POCD and potential neuroinflammation, improved postoperative recovery and clinical prognosis of patients undergoing digest tract cancer surgery. Further studies are necessary to elucidate the clinical application of DEX from an immunological perspective.

## Introduction

Systemic immune perturbations occur with cancer development (1). Tumor-burdened microenvironment affects the quantity and differentiation of T cells, neutrophils, and monocyte, especially for the elderly with underlying diseases and impaired immune function (2, 3). Radical surgery is the preferred treatment for most patients with early-stage cancer (4). However, the incidence of systemic inflammatory response syndrome (SIRS) increased during the perioperative period due to anesthesia, surgical trauma, and pre-existing comorbidities. The release of damage-associated molecular patterns (DAMPs) or alarmins following the surgical injury is the important involved mechanism (5, 6). DAMPs could activate immune cells including neutrophils and lymphocytes, and trigger the release of pro-inflammatory mediators including IL-6, IL-1, and TNF-α (7, 8). High mobility group box 1 protein (HMGB1) is a DAMP molecule. It affects the activation and differentiation of Treg and is associated with cancer recurrence and metastasis (9). Meanwhile, perioperative factors and peripheral inflammation are associated with central nervous system (CNS) neuroinflammation and pathologies (10, 11). Elevated inflammatory cytokines in the CNS are concentrated in the hippocampus, where the receptors of pro-inflammatory cytokines were highly expressed, leading to postoperative cognitive dysfunction (POCD), especially for the elderly with an impaired blood-brain barrier (BBB) (12, 13). Therefore, perioperative immune dysfunction and CNS neuroinflammation have momentous clinical implications in postoperative recovery, tumor recurrence, and metastasis, etc.

Dexmedetomidine (DEX) is a highly selective α_2_ adrenergic receptor agonist, especially for the α_2A_ adrenergic receptor located in the locus coeruleus nucleus (14). DEX has been frequently used in the perioperative period because of its sedative pharmacology. DEX could attenuate stress responses and emotional disorders, and create stable hemodynamic profiles during stressful events such as surgery or anesthetic induction (15). DEX could resemble natural sleep, increase physiological sleep-wake cycle for ICU patients, and reduce the risk of delirium (16). DEX could also reduce the level of postoperative inflammatory factors through PI3K-Akt signaling (17), and inhibit cancer development through the upregulation of miR-185 and inactivation of SOX9-Wnt-β-catenin signaling (18). Moreover, there is growing evidence that DEX has a potential role during perioperative period for the prevention and alleviation of inflammation and immune dysfunction (19).

Multiple RCTs have been conducted to determine whether perioperative intravenous DEX could alleviate postoperative SIRS and POCD in patients undergoing radical surgery (20, 21). However, due to the methodology and small sample size, interpretation of these studies has limitations and the results are inconsistent. Meanwhile, the mechanism by which DEX interferes with cellular and humoral immunity is still unclear. Therefore, this meta-analysis aimed to summarize all existing evidence and systematically review the impact of DEX on perioperative immune dysfunction, POCD, and postoperative recovery, to provide guidance for clinical treatment and prognosis.

## Methods

This meta-analysis was conducted based on the criteria of the Cochrane Handbook for Systematic Reviews of Interventions (version 6.2). The results were presented according to the preferred reporting items declared by Systematic Review and Meta-Analysis (PRISMA) 2020. Ethical approval was not required, as this study only included articles of published data in the public domain.

### Literature search

Two reviewers performed the literature search, systematically searching the PubMed, Embase, the Cochrane Library, and Web of Science databases sources until April 2022 for studies exploring the application of perioperative DEX in patients with digestive tract cancer. The following search terms were used: (1) “Dexmedetomidine”, “MPV-1440”, “Precedex” or “Dexmedetomidine Hydrochloride”, (2) “Esophageal Neoplasms”, “Stomach Neoplasms”, “Gastrointestinal Neoplasms”, “Colorectal Neoplasms”, “Colonic Neoplasms”, “Rectal Neoplasms”, “Intestinal Neoplasms”, “Esophageal Cancer”, “Gastric Cancer”, “Intestinal Cancer”, “Colorectal Cancer”, “Colon Cancer”, “Rectal Cancer”, “Gastrointestinal Cancer” or “Digestive Tract Cancer”. The above two categories of search terms were combined using the Boolean operator “and”. The search strategies are shown in **Table 1**, and the detailed electronic search strategies for PubMed, Cochrane Library, EMBASE and Web of Science databases are shown in **Supplementary Table 1**. In addition, the reference lists of the retrieved articles and prior reviews were manually checked for additional eligible studies. We applied no linguistic restrictions in the literature search.

**Table 1:** The search strategies until February 2022.

	Search terms	PubMed	Embase	Web of science	Cochrane
**#1**	Dexmedetomidine	7555	15170	9929	6184
**#2**	MPV-1440	7556	4	1	3
**#3**	Precedex	7558	523	54	82
**#4**	Dexmedetomidine Hydrochloride	7555	130	223	123
**#5**	#1 OR #2 OR #3 OR #4	7559	15170	9931	6181
**#6**	Esophageal Neoplasms	63836	2205	3419	2395
**#7**	Stomach Neoplasms	119232	5731	5952	3896
**#8**	Gastrointestinal Neoplasms	443191	1149	12158	5394
**#9**	Colorectal Neoplasms	240215	5798	13841	8733
**#10**	Colonic Neoplasms	92777	1677	3429	4402
**#11**	Rectal Neoplasms	69204	1662	4772	3351
**#12**	Intestinal Neoplasms	270675	383	3813	2216
**#13**	Esophageal Cancer	73668	35427	53390	4858
**#14**	Gastric Cancer	152596	102860	128855	8623
**#15**	Intestinal Cancer	285551	1648	49274	4676
**#16**	Colorectal Cancer	271702	233478	251089	17298
**#17**	Colon Cancer	152047	113537	158616	8164
**#18**	Rectal Cancer	76673	42414	59070	6221
**#19**	Gastrointestinal Cancer	469120	18366	118013	11785
**#20**	Digestive Tract Cancer	159,200	516	30,874	621
**#21**	#6 OR #7 OR #8 OR #9 OR #10 OR #11 OR #12 OR #13 OR #14 OR #15 OR #16 OR #17 OR #18 OR #19 OR #20	651,377	492021	964,761	56828
**#22**	#5 AND #21	92	126	97	137

### Inclusion and exclusion criteria

RCTs conducted to compare DEX with placebo in patients undergoing digest tract tumor surgery were all enrolled. Included studies need to report at least one of the outcomes, including inflammatory factors, cellular immunity, cognitive function, and prognosis. Exclusion criteria were as follows: (1) reviews, letters, editorials, or observational (prospective or retrospective cohort) study; (2) comparisons of DEX with other sedatives(midazolam, fentanyl, propofol, etc.); (3) no intravenous administration; (4) no target outcomes; (5) data was unable to obtain or insufficient. If there were overlapping data among two or more studies, we included the one with the largest sample size.

### Study selection and data abstraction

Two reviewers independently screened the titles and abstracts of the retrieved studies from the electronic databases. Subsequently, eligible studies were selected after full-text screenings according to the pre-defined criteria. Disagreements were resolved by discussion between two reviewers or consultation with the corresponding authors. The following data of the included studies were abstracted: study characteristics (first author, year of publication, and study design), study population, baseline characteristics (age, sample size, interventions, and anesthesia method), outcomes (inflammatory factors, cellular immunity, cognitive function, and prognosis), and outcome data (sample size and the number of events between groups).

### Study quality assessment

The bias risks of RCTs were assessed using the revised Cochrane risk-of-bias tool for randomized trials (RoB2), which is more detailed and comprehensive than RoB1, and includes five domains: randomization process, deviations from the intended interventions, missing outcome data, measurement of the outcome, and selection of the reported results. The level of the bias risk in each domain and overall were scored as ‘low risk’, ‘some concerns’, or ‘high risk’. We used the funnel plots to assess the publication bias, and there was no significant asymmetry in the funnel plots of the present data (**Supplementary Figures 1**–**4**). Thus, there was no significant publication bias. **Supplementary Table 2** presents the GRADE analysis of the variables examined in this meta-analysis. The certainty was high for POCD and MMSE; moderate for CRP, IL-6, IL-10, lymphocyte subsets, first flatus time, hospital stay and extubation time, and low for TNF-α.

### Statistical analysis

Statistical analysis for this meta-analysis was This meta-analysis’s statistical analyses were conducted using the Review Manager version 5.4 software (the Cochrane Collaboration 2014, Nordic Cochrane Centre Copenhagen, Denmark; https://community.cochrane.org/). The pooled effects were calculated and described by standard mean difference (SMD) with a 95% Confidence Interval (the Confidence Interval, 95% CI) and the risk ratio with 95% CI. The significant heterogeneity was indicated by a P-value of < 0.10 in the Cochrane Q test or an I² value of > 50%, which led to the use of random-effects models and the exploration of a potential source of heterogeneity. Otherwise, the fixed-effects model was selected.

## Results

### Study selection outcome

**Figure 1:**
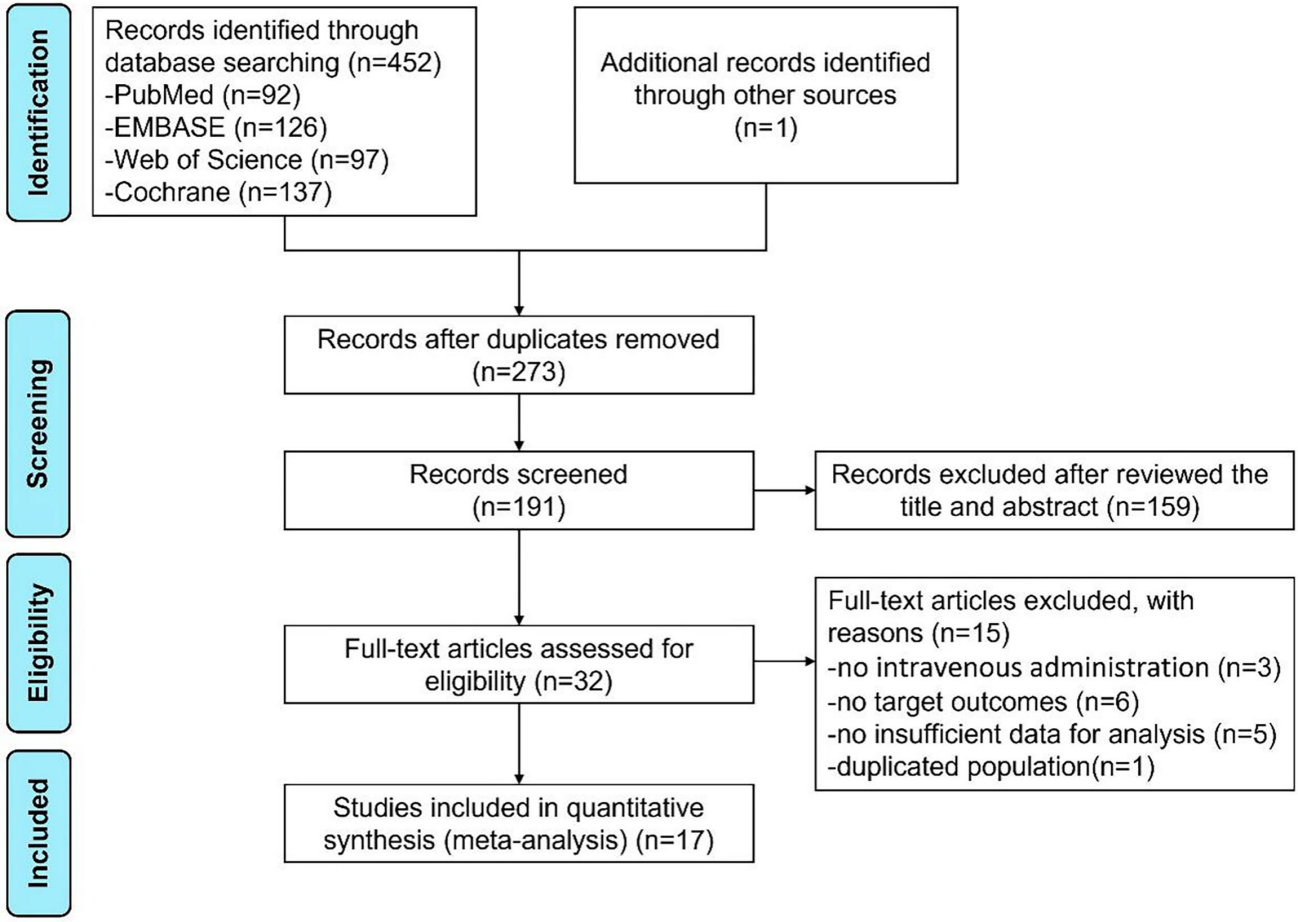
The flow chart of literature retrieval of this meta-analysis.

The flow chart of literature retrieval is shown in **Figure 1**. Through searching the PubMed, Embase, Cochrane Library, and Web of Science databases, the initial search yielded 452 articles. We also identified the potential studies by searching the reference lists of published reviews, in this way, we found a relevant article from Chinese biological and medical database. Duplicate articles were removed. After screening the records, reviewing the title and abstract, and the full-text screenings with the pre-defined criteria to exclude 15 studies, a total of 17 studies were included in this meta-analysis.

### Study characteristics

17 RCTs involving 1619 patients undergoing surgical resection of digest tract tumors were finally included. Baseline characteristics and detailed administration of included studies are shown in **Table 2**. The mean age of patients ranged from 36.3 to 74.1 years, and the sample size was from 48 to 180. The detailed usage and dosage of DEX are shown in **Table 2**. DEX was administered intravenously in all studies. 14 studies administered a loading dose before induction and followed by continuous infusion. The other 3 studies only administered a loading dose before induction. The control group was injected intravenously with the same volume of normal saline.

**Table 2:** Characteristics of included studies.

Author -Year	Trial type	Simple size		Mean Age(Years)		Male ratio (%)		Intervention study(DEX dose/administration mode)	Neoplasm’s type	Comparators	Anesthesia method
		CON	DEX	CON	DEX	CON	DEX				
**Mao Y-2020** (22)	RCT	29	29	63.5	65.2	82.8	79.3	Bolus (0.5 ug/kg) before induction, and then continuous infusion (0.2-0.4 ug/kg/h) during operation	Esophageal Cancer	Saline	I.V.
**Dong W-2017** (23)	RCT	37	37	38.7	36.3	54.1	62.2	Bolus (1 ug/kg; 15 min) before induction, and then continuous infusion (0.2 ug/kg/h) during operation	Gastric Cancer	Saline	Combined
**Niu Y-2022**	RCT	30	30	69.0	68.0	60.0	56.7	Bolus (0.6 ug/kg; 15 min) before induction, and then continuous infusion (0.2 ug/kg/h) during operation	Gastric Cancer	Saline	I.V.
**Wang Z-2020** (17)	RCT	50	60	68.3	68.4	60.0	63.3	Bolus (0.5 ug/kg) before induction, and then continuous infusion (0.4 ug/kg/h) during operation	Gastric Cancer	Saline	Combined
**Zhu Z-2017** (24)	RCT	45	45	51.5	51.8	55.6	48.9	Bolus (0.6 ug/kg) before induction	Gastric Cancer	Saline	Combined
**Huai Q-2022** (25)	RCT	40	40	63.5	61.7	65.0	60.0	Bolus (0.5 ug/kg; 10 min) before induction, and then continuous infusion (0.4 ug/kg/h) during operation	Colon Cancer	Saline	Inhalation
**Wang K-2018** (26)	RCT	69	72	45.3	42.5	47.8	50.0	Bolus (1 ug/kg; 10-15 min) before induction, and then continuous infusion (1 ug/kg/h) during operation	Colon Cancer	Saline	Combined
**Zhao L-2020** (27)	RCT	84	92	53.1	52.5	56.0	45.7	Bolus (200ug)	Colon Cancer	Saline	I.V.
**Ben Z-2016** (28)	RCT	44	44	68.6		59.1		Bolus (0.5 ug/kg) before induction, and then continuous infusion (0.1 ug/kg/h) during operation	Rectal Cancer	Saline	I.V.
**Chen C-2016** (29)	RCT	30	30	60.1	56.7	50.0	46.7	Bolus (1 ug/kg; 10 min) before induction, and then continuous infusion (0.3 ug/kg/h) during operation	Colorectal Cancer	Saline	Inhalation
**Kong Y-2021** (30)	RCT	60	120	65.0	65.0	NA	NA	Bolus (0.5 ug/kg) before induction, and then continuous infusion (0.4/0.8 ug/kg/h) during operation	Colorectal Cancer	Saline	I.V.
**Liu X-2017** (31)	RCT	48	48	69.1	68.4	54.2	52.1	Bolus (1.5 ug/kg;30min)	Colorectal Cancer	Saline	I.V.
**Liu Y-2020** (32)	RCT	24	24	68.6	69.6	54.2	62.5	Bolus (0.5 ug/kg; 15 min) before induction, and then continuous infusion (0.6 ug/kg/h) during operation	Colorectal Cancer	Saline	I.V.
**Pan C-2016** (33)	RCT	41	41	73.9	71.9	NA	48.8	Bolus (0.5 ug/kg) before induction, and then continuous infusion (0.3 ug/kg/h) during operation	Colorectal Cancer	Saline	NA
**Sun W-2021** (34)	RCT	28	28	59.0	60.0	60.7	67.9	Bolus (1 ug/kg; 10 min) before induction, and then continuous infusion (0.5 ug/kg/h) during operation	Colorectal Cancer	Saline	Combined
**Zhang J-2019** (35)	RCT	60	80	74.1	73.8	66.7	63.8	Bolus (1 ug/kg; 15 min) before induction, and then continuous infusion (0.2-0.7 ug/kg/h) during operation	Colorectal Cancer	Saline	I.V.
**Zhang Y-2014** (36)	RCT	20	60	71.5	72.0	55.2	60.0	Bolus (0.5 ug/kg; 15 min) before induction, and then continuous infusion (0.2/0.5/0.8 ug/kg/h) during operation	Colorectal Cancer	Saline	I.V.

### Risk of bias assessment

**Figure 2:**
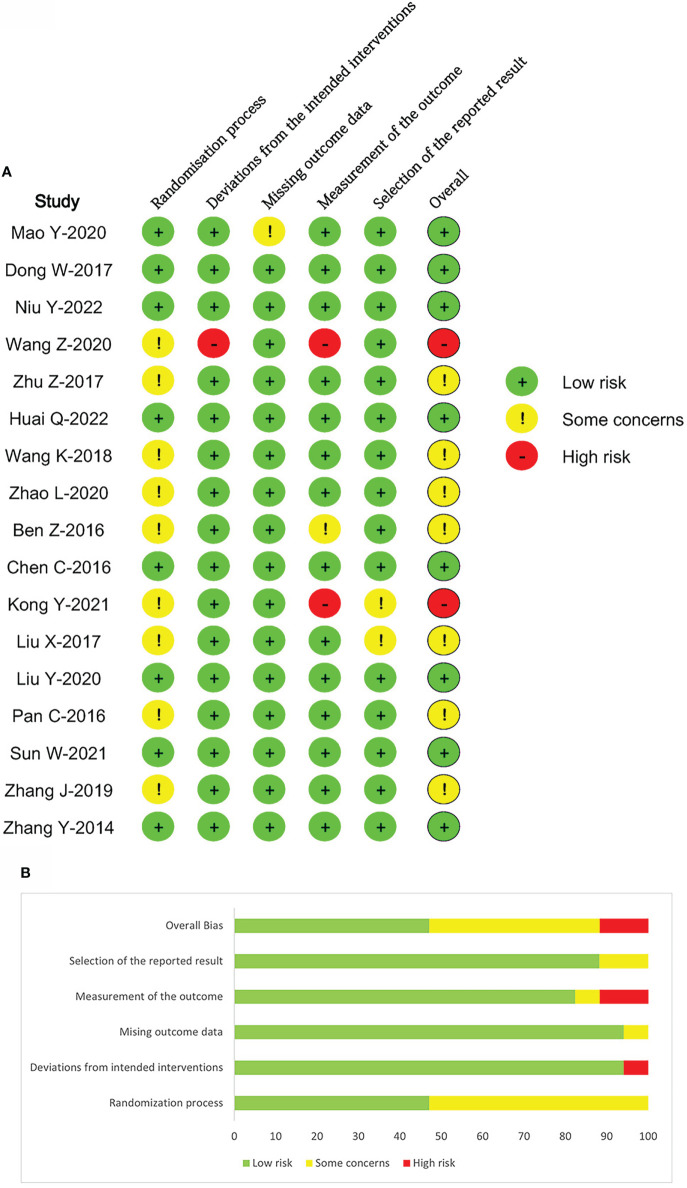
Methodological quality graph and summary of the included studies: Risk of bias summary **(A)**, Risk of bias graph **(B)**.

The risk of bias assessment for individual studies is shown in **Figure 2**. Of the included trials, nine studies did not give a specific randomization process, which was categorized as “some concerns”. One study deviated from the intended intervention, and two studies were judged high risk in terms of the measurement of the outcomes. The remaining eight studies were identified as low risk.

### Synthesis of results

The different outcomes of 17 studies were synthesized. First, the changes of inflammatory mediators were concerned, as surgical and anesthetic factors can lead to systemic inflammatory responses that further exacerbate central inflammation. Then, T lymphocyte subsets were observed, which has been proven to be an important indicator of immune dysfunction, The systemic inflammatory response and immune dysfunction in the perioperative period will further aggravate the CNS neuroinflammation and cognitive dysfunction of patients, thus affecting postoperative recovery and clinical prognosis. Therefore, our study comprehensively considered the effects of DEX on inflammatory mediators, T lymphocytes, POCD, and postoperative recovery.

### Effects of DEX on postoperative inflammatory mediators

**Figure 3:**
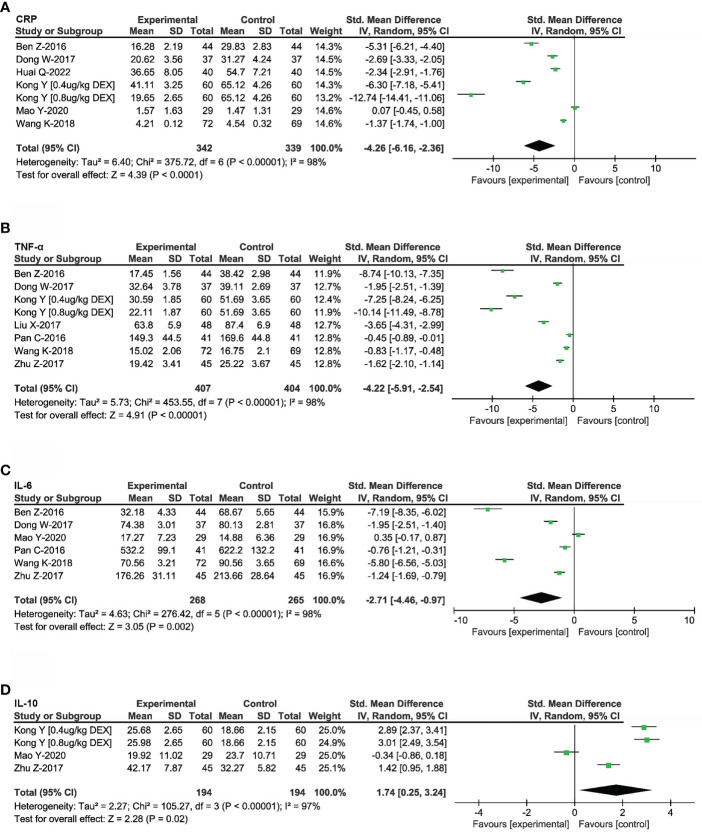
Effects of DEX on inflammatory mediators. Forest plot of odds ratio, analyzed by Mantel-Haenszel statistics in the random-effect model. Meta-analysis of the DEX effect on CRP **(A)**, TNF-a **(B)**, IL-6 **(C)** and IL-10 **(D)** respectively.

9 RCTs that reported the postoperative levels of inflammatory mediators in 889 patients with digest tract tumors were included (22–26, 28, 30, 31, 33). CRP has been used as a marker of acute inflammatory responses in a variety of psychiatric and physical conditions. As shown in **Figure 3A**, the pooled results based on the random-effects model indicated that the use of DEX was significantly associated with reduced CRP release (SMD = -4.26, 95%CI: -6.16, -2.36). Meanwhile, in order to illustrate the effects of DEX on inflammation, several inflammatory cytokines were investigated based on the involved studies. The results indicated that DEX decreased the release of TNF-α (SMD = -4.22, 95%CI: -5.91, -2.54, **Figure 3B**) and IL-6 (SMD = -2.71, 95%CI: -4.46, -0.97, **Figure 3C**), but increased the release of IL-10 (SMD = 1.74, 95%CI: 0.25, 3.24, **Figure 3D**). These release changes could improve cellular immunosuppression and attenuate the progress of digest tract cancer.

### Effects of DEX on postoperative T lymphocytes

**Figure 4:**
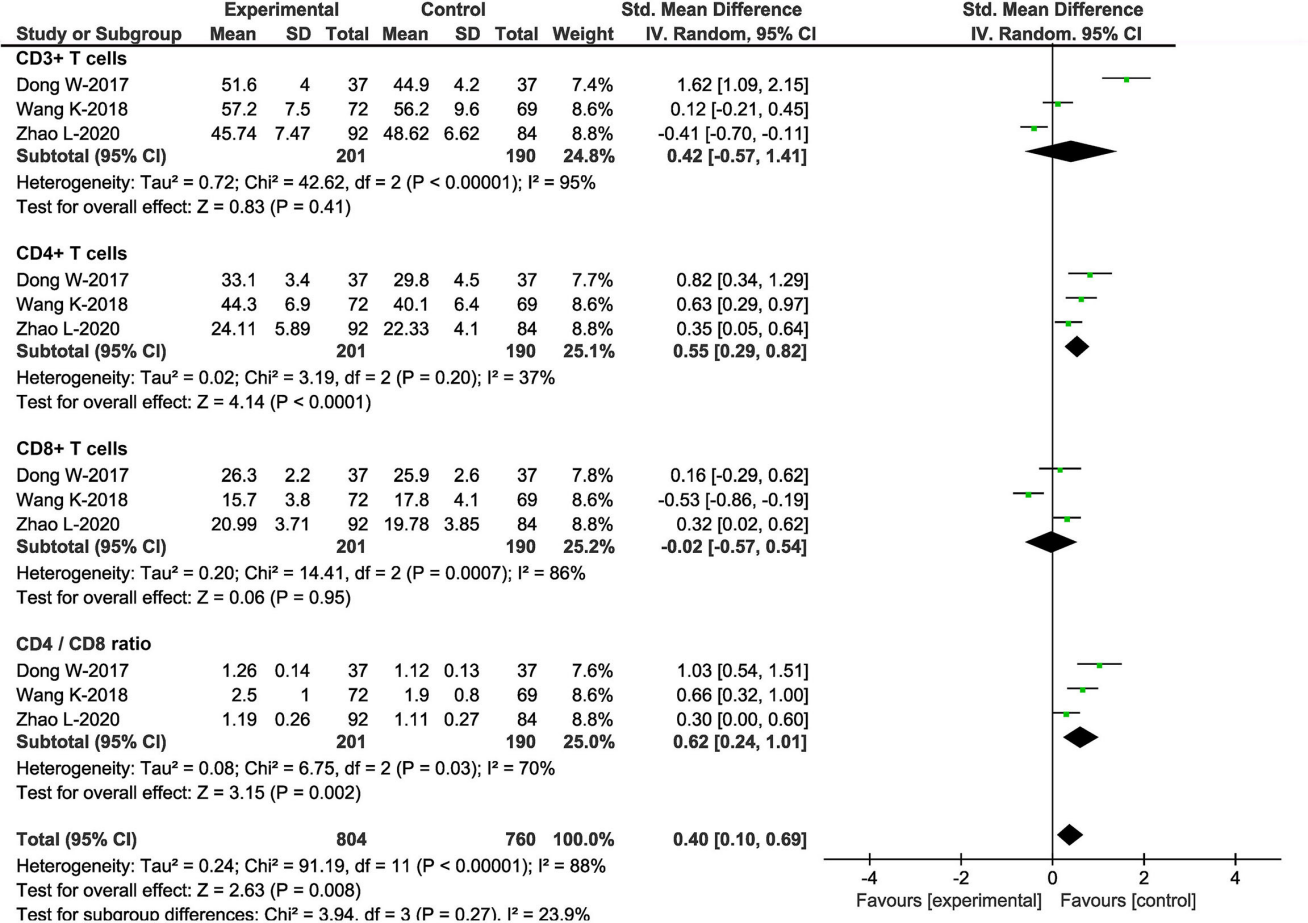
Effects of DEX on T lymphocytes. Forest plot of odds ratio, analyzed by Mantel-Haenszel statistics in the random -effect model. Meta-analysis of the DEX effect on CD3+ T cells, CD4+ T cells, CD8+ T cells and CD4+ /CD8+ ratio respectively.

T lymphocytes play roles in perioperative immune homeostasis and tumor resistance within the digestive tract; thus, the state and subsets of T lymphocytes were investigated. Three studies, including 391 patients, investigated the effects of DEX on T lymphocytes (23, 26, 27). T lymphocyte subsets included were CD3+, CD4+, and CD8+ T cells. At the same time, we calculated the CD4+/CD8+ ratio, which decreased and indicated impaired immune functions and poor prognosis. Separately, there was no significant difference in the counts of CD3+ T cells (SMD = 0.42, 95%CI: -0.57, 1.41) and CD8+ T cells (SMD = -0.02, 95%CI: -0.57, 0.54) between patients treated with and without DEX at 24h postoperatively. In contrast, CD4+ T cell counts (SMD = 0.55, 95%CI: 0.29, 0.82) and CD4+/CD8+ ratio (SMD = 0.62, 95%CI: 0.24, 1.01) increased in patients with DEX (**Figure 4**). Therefore, DEX attenuated the variation of cellular immune functions caused by surgical trauma, stress responses and other perioperative factors.

### Effects of DEX on cognitive function

**Figure 5:**
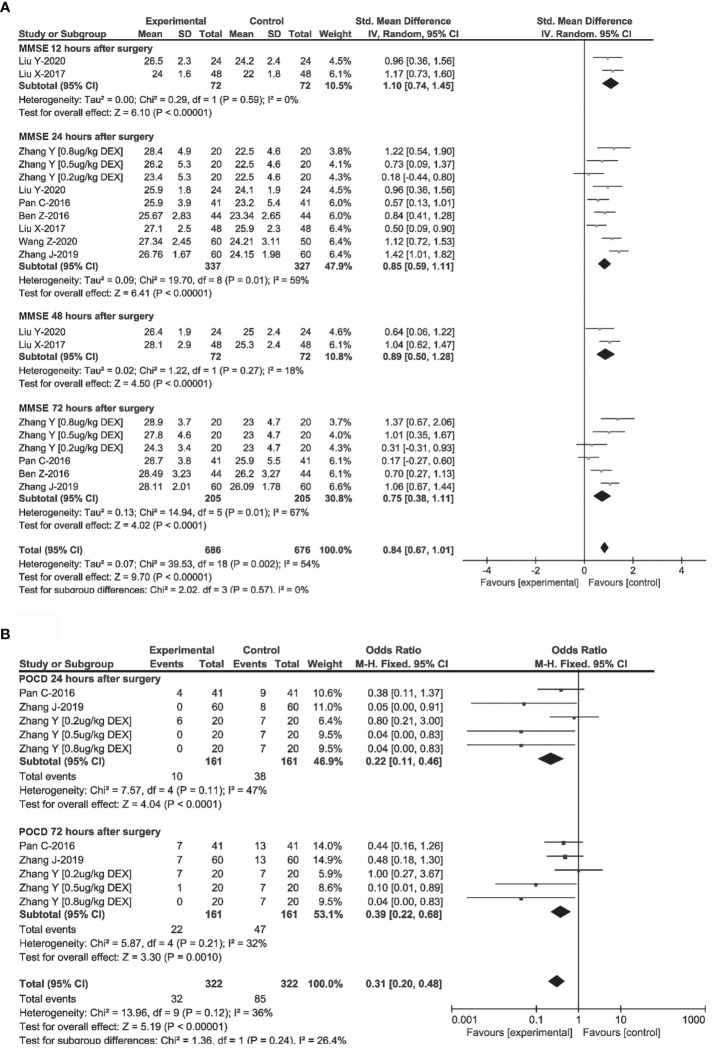
Effects of DEX on postoperative cognitive function. Forest plot of odds ratio, analyzed by Mantel-Haenszel statistics in the random-effect model. Meta-analysis of the DEX effect on MMSE at 24h, 48h, 72h after surgery **(A)**, and the occurrence of POCD at 24h, 72h after surgery **(B)** respectively.

Random-effects model and fixed-effect model were used to synthesize the MMSE scores and the incidence of POCD at different time points after surgery in 7 RCTs to consider the effect of DEX on postoperative cognitive function comprehensively (17, 28, 31–33, 35, 36). As shown in **Figure 5**, in digest tract tumor patients, DEX administration was associated with higher MMSE scores at 12h (SMD = 1.10, 95%CI: 0.74, 1.45), 24h (SMD = 0.85, 95%CI: 0.59, 1.11), 48h (SMD = 0.89, 95%CI: 0.50, 1.28) and 72h (SMD = 0.75, 95%CI: 0.38, 1.11) after surgery. DEX administration was also associated with a significant reduction in the occurrence of POCD at 24h (OR = 0.22, 95%CI: 0.11, 0.46) and 72h (OR = 0.39, 95%CI: 0.22, 0.68) after surgery. As previous studies indicated, the changes of cognitive function could be associated with perioperative immune function and neuroinflammation (37).

### Effects of DEX on postoperative recovery

**Figure 6:**
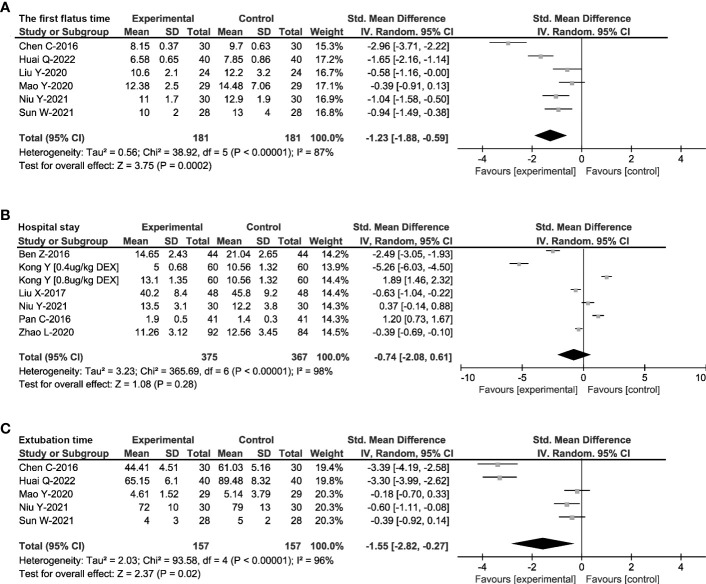
Effects of DEX on prognosis. Forest plot of odds ratio, analyzed by Mantel-Haenszel statistics in the random-effect model. Meta-analysis of the effect on the first flatus time **(A)**, hospital stay **(B)** and postoperative extubation time **(C)** respectively.

The perioperative stress and inflammation could affect gastrointestinal motility, extubation time and hospital stay, which are considered as the indicators for patient recovery and clinical prognosis. Therefore, a random-effects model was used to synthesize the postoperative extubation time, first flatus time and hospital stay of 808 patients in 11 RCTs (22, 25, 27–34, 38). As shown in **Figure 6**, DEX administration decreased first flatus time (SMD = -1.55, 95%CI: -2.82, -0.27), and the length of hospital stay (SMD = -1.23, 95%CI: -1.88, -0.59). However, there was no significant difference in postoperative extubation time (SMD = -0.74, 95%CI: -2.08, 0.61).

## Discussion

This study investigated the effects of DEX administration on postoperative immune dysfunction, cognitive function, and recovery. The results indicated that DEX decreased the level of CRP, TNF-α, and IL-6, and increased the level of IL-10. DEX increased CD4+ T cell counts and CD4+/CD8+ ratio. Furthermore, DEX led to higher MMSE scores during postoperative periods and a significant reduction in the occurrence of POCD. DEX decreased the first flatus time and the length of hospital stay, but not postoperative extubation time. Therefore, DEX administration attenuated postoperative systemic inflammatory response and immune dysfunction, improved cognitive function, and recovery in patients undergoing digest tract cancer surgery.

Previous studies showed that cancer could lead to systemic immune perturbations and affect responses to new immune challenges (1). Meanwhile, surgery could put body in a stress state, which leads to the imbalance of the neuro-endocrine-immune network, resulting in low cellular and humoral immune functions (39). The present results indicated that DEX could decrease postoperative levels of CRP, TNF-α and IL-6, and increase the level of IL-10. CRP is widely used as a marker of inflammation, infection, and tissue damage (40), and plays an important role in tumor development. The prognostic value of CRP has already been shown for digestive tract cancer (41). As a major cytokine in the acute phase, IL-6 is associated with the pathological progress of digest tract cancer (42). Low serum IL-6 level has been shown to be an independent prognostic factor for disease-free survival of patients with hepatitis B virus-related hepatocellular carcinoma who underwent hepatic resection (43). IL-10 is an anti-inflammatory mediator and plays a dual role in immune modulation, depending on cell type and environment (44). The immunosuppressive role of IL-10 has led to the general view that its presence during the development of cancer would facilitate tumor immune escape (45). However, A recent study showed that IL-10 potentiated the antitumor activity of CD8+ T cells by increasing its tissue infiltration, inducing IFN-γ, and favoring effective T cell memory responses (46, 47). Previous studies showed that surgery triggered a central response *via* afferent nerves to activate the sympathetic-adrenal-medullary (SAM) axis, and increased blood leukocyte counts (48, 49). Dexmedetomidine could affect SAM, reduce the release of epinephrine and norepinephrine, and decrease inflammatory factors (50, 51). DEX could also reverse HMGB1 related systemic and hippocampal inflammatory responses through the following mechanisms: 1) stimulation of the vagus nerve, 2) elimination of DAMP molecules and damaged mitochondria through PINK1-mediated mitophagy, 3) promotion the resolution of inflammation through TGF-31 secreted by F4/80Ly6G (52–54). Therefore, DEX could attenuate the negative effects of DAMP and inflammatory responses during digest tract cancer surgery.

T-lymphocyte could maintain the homeostasis within digestive tract mucosa and play an important role in anti-tumor immunity. The present results indicated that DEX could increase postoperative CD4+ T cells and CD4+/CD8+ ratio. CD4+ T cells play an important role in anti-tumor response. Recent studies have shown that CD4+ T cells not only enhanced the tumoricidal activity of other anti-tumor effector cells, but also blocked tumor growth through directly affecting the progression of tumor cell cycle (55). All mature peripheral T-lymphocytes, labeled by CD3+, represent the general level of immunity, and reduction of CD4+/CD8+ ratio indicates decreased immune function and poor prognosis (56, 57). Therefore, DEX could attenuate perioperative cellular immune function suppression, and further alleviate inflammatory response.

The patients with cancer are more vulnerable to postoperative systemic immune dysfunction and the peripheral environment is connected with CNS (58). Pro-inflammatory signals from peripheral immune system could enter CNS and cause neurotoxic symptoms (59). During radical surgery, intracellular substances released from damaged tissues and organs will be recognized by immune cells (60). Immune cells affect the expression of inflammatory factors, which can trigger the CNS response and amplify neuroinflammation through vagal afferents or BBB (61, 62). The inflammatory cells in CNS release more inflammatory cytokines which concentrate in specific brain regions, leading to the occurrence of POCD (37, 63, 64) and resulting in the development of neurodegenerative diseases (65). The present results indicated that DEX decreased the occurrence of POCD, which could be related to the effect of DEX on perioperative immune function and potential neuroinflammation.

Surgery is a common treatment for gastrointestinal tumors. The digestive tract is an important immune organ, and surgery could cause irreversible damage to it. Resection of digest tract cancer leads to anatomical abnormality and deficient intestinal function, and pneumoperitoneum induces ischemia and hypoxia in intestinal mucosa, which impair intestinal mucosa barrier function and result in intestinal bacterial translocation and inflammatory responses (56, 66). Meanwhile, advanced age, malnutrition, co-morbidities, and the occurrence of POCD could also affect the recovery of gastrointestinal motility, length of hospital stay and even the prognosis of patients. The present result indicated that DEX could decrease first flatus time and the length of hospital stay. The potential mechanisms for flatus time reduction include: 1) DEX reduces surgical stress and pain, then improves hemodynamics stability and intestinal microcirculation alteration (67), 2) DEX improves cognitive dysfunction and early postoperative activity; 3) DEX accelerates intestinal wound healing through increasing intestinal epithelial cell proliferation (68). Postoperative systemic immune dysfunction, POCD and gastrointestinal dysfunction lead to an array of symptoms, and other physiological/psychological diseases. They could affect the length of hospital stay, and the standard of living after discharge. Therefore, DEX administration could be valuable strategy for the patients with tumors to improve postoperative gastrointestinal function and prognosis.

This meta-analysis describes the effect of dexmedetomidine on postoperative systemic inflammation and recovery from the levels of immunomodulators, cellular immunity, cognitive function and prognosis. Hence, the coverage is more comprehensive. Meanwhile, the practical and precise strategies used for comprehensive searches of four databases, inclusion and exclusion criteria, and consideration of study quality indicated the stability and robustness of the present meta-analysis. At the same time, the meta-analysis has some limitations. Firstly, variations in the types and duration of surgery, inconsistent baseline data, concentration and duration of DEX administration may contribute the heterogeneity among studies. However, the funnel plots showed no significant asymmetry, indicating acceptable heterogeneity, as well as the stability and robustness of this meta-analysis. Secondly, the two included studies didn’t describe the detailed blinding process in the methods, leading to the suspicion that patients and investigators were aware of the experimental groups. Then the two studies were identified as high risk in the bias assessment. Thirdly, the RCTs included in this meta-analysis covered a long-time span, in which the surgical methods and equipment may have changed. All these factors may lead to instability in the present analysis. Finally, this meta-analysis has not been pre-registered in a protocol (eg. in PROSPERO), which may result in potential bias. Thus, more prospective studies with larger samples sizes and standardized protocols are required in the future to accurately determine the effects of DEX in postoperative systemic inflammation.

In conclusion, the present study found that DEX administration attenuated postoperative systemic inflammatory response and immune dysfunction. Then, DEX decreased the occurrence of POCD, the first flatus time and length of hospital stay of the patients undergoing digest tract cancer surgery. These results provided a potential therapeutic strategy to improve perioperative immune function, CNS function and clinical prognosis of digest tract cancer. Further studies are necessary to elucidate the clinical application of DEX from an immunological perspective.

## Data availability statement

The original contributions presented in the study are included in the article/**Supplementary Material**. Further inquiries can be directed to the corresponding author.

## Author contributions

WX and YuZ collected and performed the data extraction, and wrote the manuscript. ZS and SL contributed to data analysis. KF and YeZ contributed to study design and data analysis. YW, CL, and MZ coordinated data collection and manuscript revision. HZ and ZZ contributed to study design and manuscript revision. CN conceptualized and designed the study, supervised data collection, drafted and revised the manuscript. All authors approved the submitted version.

## Funding

This work was supported by Beijing Hope Run Special Fund of Cancer Foundation of China (No. LC2020A01), the National Natural Science Foundation of China (Nos. 82171195, 81771146, 82101281), and Talent Project of National Cancer Center/Cancer Hospital Chinese Academy of Medical Sciences (For CN).

## Conflict of interest

The authors declare that the research was conducted in the absence of any commercial or financial relationships that could be construed as a potential conflict of interest.

## Publisher’s note

All claims expressed in this article are solely those of the authors and do not necessarily represent those of their affiliated organizations, or those of the publisher, the editors and the reviewers. Any product that may be evaluated in this article, or claim that may be made by its manufacturer, is not guaranteed or endorsed by the publisher.

## Supplementary material

Supplementary Table 1 GRADE Summary of findings for inflammatory mediators, T lymphocytes, cognitive function and prognosis. a. Most information from studies at low or moderate risk of bias (one study at high risk of bias). 1. rate down for inconsistency due to high heterogeneity. 2. rate down for imprecision due to wide CIs.

Supplementary Table 2 Detailed search strategy terms for PubMed, Cochrane Library, EMBASE and Web of Science databases.

Supplementary Figure 1 The funnel plots were used to assess the publication bias for the STD Mean Difference of the DEX effects on CRP **(A)**, TNF-a **(B)**, IL-6 **(C)** and IL-10 **(D)**.

Supplementary Figure 2 The funnel plots were used to assess the publication bias for the STD Mean Difference of the DEX effects on CD3+ T cells, CD4+ T cells, CD8+ T cells and CD4/CD8 ratio respectively.

Supplementary Figure 3 The funnel plots were used to assess the publication bias for the STD Mean Difference of the DEX effects on MMSE at 24h, 48h, 72h after surgery **(A)**, and the occurrence of POCD at 24h, 72h after surgery **(B)**.

Supplementary Figure 4 The funnel plots were used to assess the publication bias for the STD Mean Difference of the DEX effects on the first flatus time **(A)**, hospital stay **(B)** and postoperative extubation time **(C)**.
