# Supplementary material for: Effect of dexmedetomidine on postoperative systemic inflammation and recovery in patients undergoing digest tract cancer surgery: A meta-analysis of randomized controlled trials
Source: Front Oncol. 2022 Sep 14;12:970557. doi: 10.3389/fonc.2022.970557 (PMC9518820; doi:10.3389/fonc.2022.970557)
Supplement: Supplementary Table 1 — GRADE Summary of findings for inflammatory mediators, T lymphocytes, cognitive function and prognosis. a. Most information from studies at low or moderate risk of bias (one study at high risk of bias). 1. rate down for inconsistency due to high heterogeneity. 2. rate down for imprecision due to wide CIs. [file Table_1.pdf]

Supplementary table 1 GRADE Summary of findings for inflammatory mediators, T lymphocytes, cognitive function and prognosis

Certainty assessment							Summary of findings		
Participants (studies)	Risk of bias	Inconsistency	Indirectness	Imprecision	Publication bias	Overall certainty of evidence	Study event rates (%)		Relative effect [MD or RR (95% CI)] with dexmedetomidine
							With no DEX	With DEX	
681 for CRP (7 RCTs)	serious [a]	serious	not serious	not serious	none	⊕⊕⊕○ Moderate[1]	339	342	SMD 4.26 lower (6.16 lower to 2.36 lower)
811 for TNF-α (8 RCTs)	serious [a]	serious	not serious	serious	none	⊕⊕○○ Low[1,2]	404	407	SMD 4.22 lower (5.91 lower to 2.54 lower)
533 for IL-6 (6 RCTs)	not serious	serious	not serious	not serious	none	⊕⊕⊕○ Moderate[1]	268	265	SMD 2.71 lower (4.46 lower to 0.97 lower)
388 for IL-10 (4 RCTs)	serious [a]	serious	not serious	not serious	none	⊕⊕⊕○ Moderate[1]	194	194	SMD 1.74 higher (0.25 higher to 3.24 higher)
1564 for Lymphocyte subsets (3 RCTs)	not serious	serious	not serious	not serious	none	⊕⊕⊕○ Moderate[1]	760	804	SMD 0.4 higher (0.1 higher to 0.69 higher)
1362 for MMSE (9 RCTs)	serious [a]	not serious	not serious	not serious	none	⊕⊕⊕⊕ High	676	686	SMD 0.84 higher (0.67 higher to 1.01 higher)
410 for POCD (6 RCTs)	not serious	not serious	not serious	not serious	none	⊕⊕⊕⊕ High	32/322	85/322	OR 0.31 (0.20 to 0.48)
362 for First flatus time (6 RCTs)	not serious	serious	not serious	not serious	none	⊕⊕⊕○ Moderate[1]	181	181	SMD 1.23 lower (1.88 lower to 0.59 lower)

742 for Hospital stay (7 RCTs)	serious [a]	serious	not serious	not serious	none	⊕⊕⊕○ Moderate[1]	367	375	SMD 1.55 lower (2.82 lower to 0.27 lower)
314 for Extubation time (5 RCTs)	not serious	serious	not serious	not serious	none	⊕⊕⊕○ Moderate[1]	157	157	SMD 0.74 lower (2.08 lower to 0.61 higher)

a. Most information from studies at low or moderate risk of bias (one study at high risk of bias)

1. rate down for inconsistency due to high heterogeneity

2. rate down for imprecision due to wide CIs
