# Supplementary material for: Effect of dexmedetomidine on postoperative systemic inflammation and recovery in patients undergoing digest tract cancer surgery: A meta-analysis of randomized controlled trials
Source: Front Oncol. 2022 Sep 14;12:970557. doi: 10.3389/fonc.2022.970557 (PMC9518820; doi:10.3389/fonc.2022.970557)
Supplement: Supplementary Table 2 — Detailed search strategy terms for PubMed, Cochrane Library, EMBASE and Web of Science databases. [file Table_2.pdf]

Supplementary table 2 Detailed search strategy terms for PubMed, Cochrane Library, EMBASE and Web of Science databases

Search strategy terms for PubMed, Cochrane Library, EMBASE and Web of Science databases.
The PubMed search terms were as follows: for 92 studies<br>#1 ((((("Dexmedetomidine"[Mesh]) OR (MPV-1440[Title/Abstract])) OR (MPV 1440[Title/Abstract])) OR (MPV1440[Title/Abstract])) OR (Precedex[Title/Abstract])) OR (Dexmedetomidine Hydrochloride[Title/Abstract])) OR (Hydrochloride, Dexmedetomidine[Title/Abstract])<br>#2 (((((((Esophageal Neoplasms[Mesh]) OR (Stomach Neoplasms[Title/Abstract])) OR (Gastrointestinal Neoplasms[Title/Abstract])) OR (Colorectal Neoplasms[Title/Abstract])) OR (Colonic Neoplasms[Title/Abstract])) OR (Rectal Neoplasms[Title/Abstract])) OR (Intestinal Neoplasms[Title/Abstract]))<br>#3 ((((((((Esophageal Cancer[Title/Abstract])) OR (Gastric Cancer[Title/Abstract]) OR (Intestinal Neoplasms[Title/Abstract]) OR (Colorectal Cancer[Title/Abstract]) OR (Colon Cancer[Title/Abstract]) OR (Rectal Cancer[Title/Abstract]) OR (Gastrointestinal Cancer[Title/Abstract]) OR (Digestive Tract Cancer[Title/Abstract])<br>#4 #2 OR #3<br>#5 #1 AND #4
The Cochrane search terms were as follows: for 137 studies<br>#1 MeSH descriptor: [Dexmedetomidine] explode all trees<br>#2 MeSH descriptor: [MPV-1440] explode all trees<br>#3 MeSH descriptor: [Precedex] explode all trees<br>#4 MeSH descriptor: [Dexmedetomidine Hydrochloride] explode all trees<br>#5 #1 OR #2 OR #3 OR #4<br>#6 MeSH descriptor: [Esophageal Neoplasms] explode all trees<br>#7 MeSH descriptor: [Stomach Neoplasms] explode all trees<br>#8 MeSH descriptor: [Gastrointestinal Neoplasms] explode all trees<br>#9 MeSH descriptor: [Colorectal Neoplasms] explode all trees<br>#10 MeSH descriptor: [Colonic Neoplasms] explode all trees<br>#11 MeSH descriptor: [Rectal Neoplasms] explode all trees<br>#12 MeSH descriptor: [Intestinal Neoplasms] explode all trees<br>#13 MeSH descriptor: [Esophageal Cancer] explode all trees<br>#14 MeSH descriptor: [Gastric Cancer] explode all trees<br>#15 MeSH descriptor: [Intestinal Cancer] explode all trees<br>#16 MeSH descriptor: [Colorectal Cancer] explode all trees<br>#17 MeSH descriptor: [Colon Cancer] explode all trees<br>#18 MeSH descriptor: [Rectal Cancer] explode all trees<br>#19 MeSH descriptor: [Gastrointestinal Cancer] explode all trees<br>#20 MeSH descriptor: [Digestive Tract Cancer] explode all trees<br>#21 MeSH descriptor: #6 OR #7 OR #8 OR #9 OR #10 OR #11 OR #12 OR #13 OR #14 OR #15 OR #16 OR #17 OR #18 OR #19 OR #20<br>#22 #5 AND #21
The EMBASE search terms were as follows: for 126 studies<br>#1 Dexmedetomidine:ab,ti<br>#2 'Esophageal Neoplasms':ab,ti OR 'Stomach Neoplasms':ab,ti OR 'Gastrointestinal Neoplasms':ab,ti OR 'Colorectal Neoplasms':ab,ti OR 'Colonic Neoplasms':ab,ti OR 'Rectal Neoplasms':ab,ti OR 'Intestinal Neoplasms':ab,ti OR 'Esophageal Cancer':ab,ti OR 'Gastric Cancer':ab,ti OR 'Intestinal Cancer':ab,ti OR 'Colorectal Cancer':ab,ti OR 'Colon Cancer':ab,ti OR 'Rectal Cancer':ab,ti OR 'Gastrointestinal Cancer':ab,ti OR 'Digestive

	Tract Cancer':ab,ti
#3	#1 AND #2
The Web of Science search terms were as follows: for 97 studies	
#1	TS=(Dexmedetomidine OR MPV-1440 OR MPV 1440 OR MPV1440 OR Precedex OR Dexmedetomidine Hydrochloride OR Hydrochloride, Dexmedetomidine)
#2	TS=(Esophageal Neoplasms OR Stomach Neoplasms OR Gastrointestinal Neoplasms OR Colorectal Neoplasms OR Colonic Neoplasms OR Rectal Neoplasms OR Intestinal Neoplasms)
#3	TS=(Esophageal Cancer OR Gastric Cancer OR Intestinal Neoplasms OR Colorectal Cancer OR Colon Cancer OR Rectal Cancer OR Gastrointestinal Cancer OR Digestive Tract Cancer)
#4	#2 OR #3
#5	#1 AND #4
