# Supplementary figures and images for: Effect of dexmedetomidine on postoperative systemic inflammation and recovery in patients undergoing digest tract cancer surgery: A meta-analysis of randomized controlled trials
Source: Front Oncol. 2022 Sep 14;12:970557. doi: 10.3389/fonc.2022.970557 (PMC9518820; doi:10.3389/fonc.2022.970557)

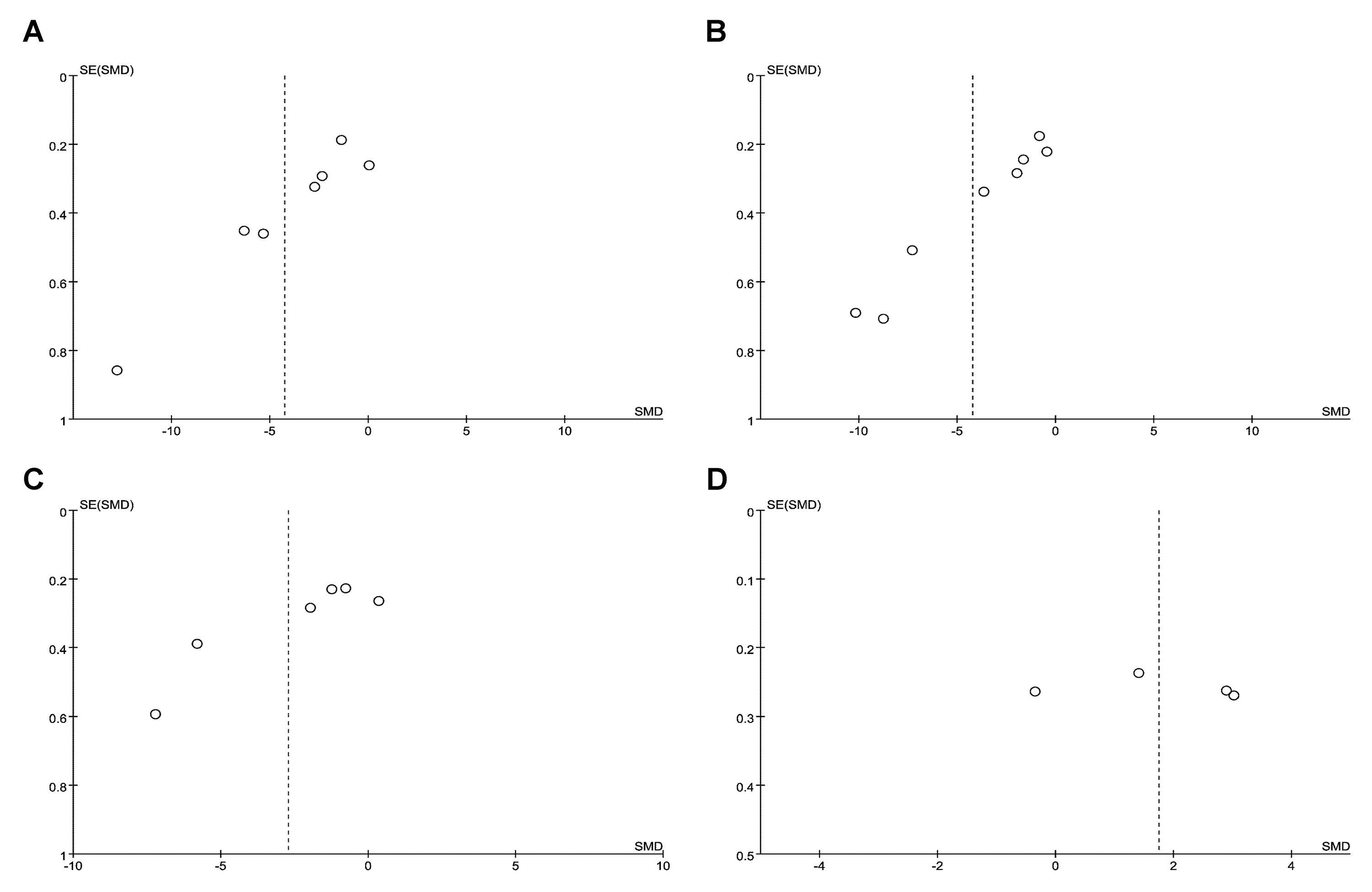

Supplement: Supplementary Figure 1 — The funnel plots were used to assess the publication bias for the STD Mean Difference of the DEX effects on CRP (A), TNF-a (B), IL-6 (C) and IL-10 (D). [file Image_1.jpeg]

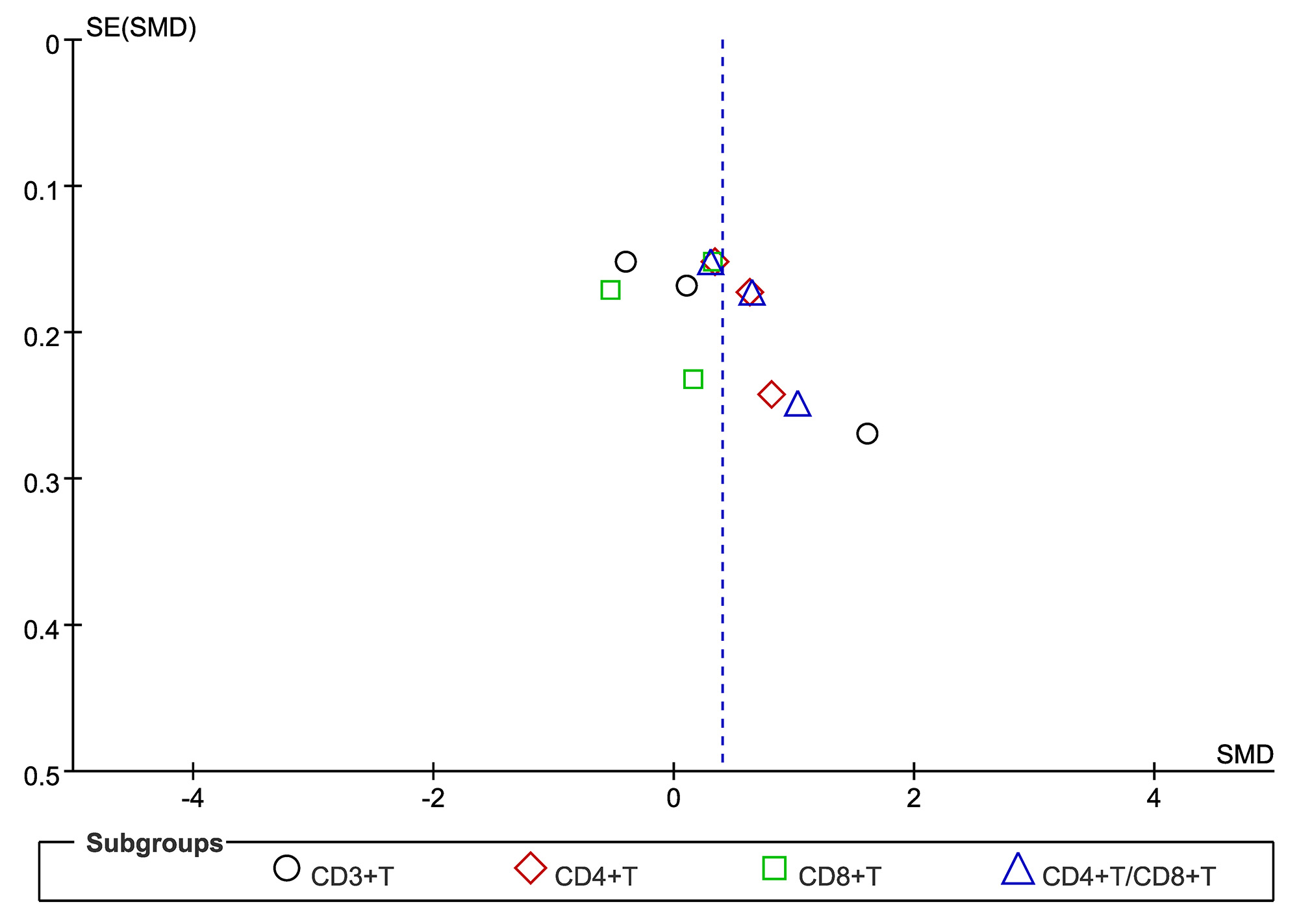

Supplement: Supplementary Figure 2 — The funnel plots were used to assess the publication bias for the STD Mean Difference of the DEX effects on CD3+ T cells, CD4+ T cells, CD8+ T cells and CD4/CD8 ratio respectively. [file Image_2.jpeg]

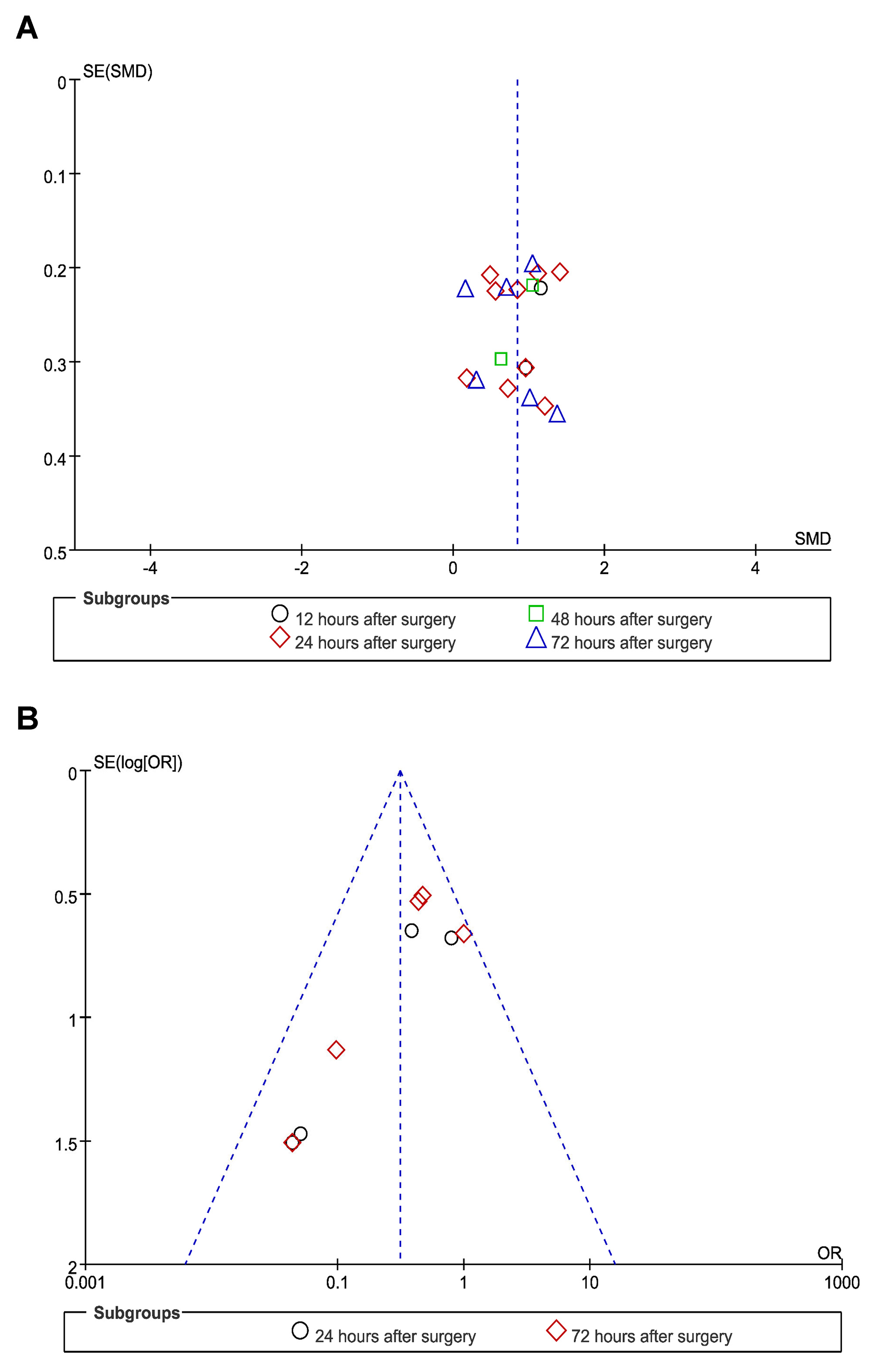

Supplement: Supplementary Figure 3 — The funnel plots were used to assess the publication bias for the STD Mean Difference of the DEX effects on MMSE at 24h, 48h, 72h after surgery (A), and the occurrence of POCD at 24h, 72h after surgery (B). [file Image_3.jpeg]

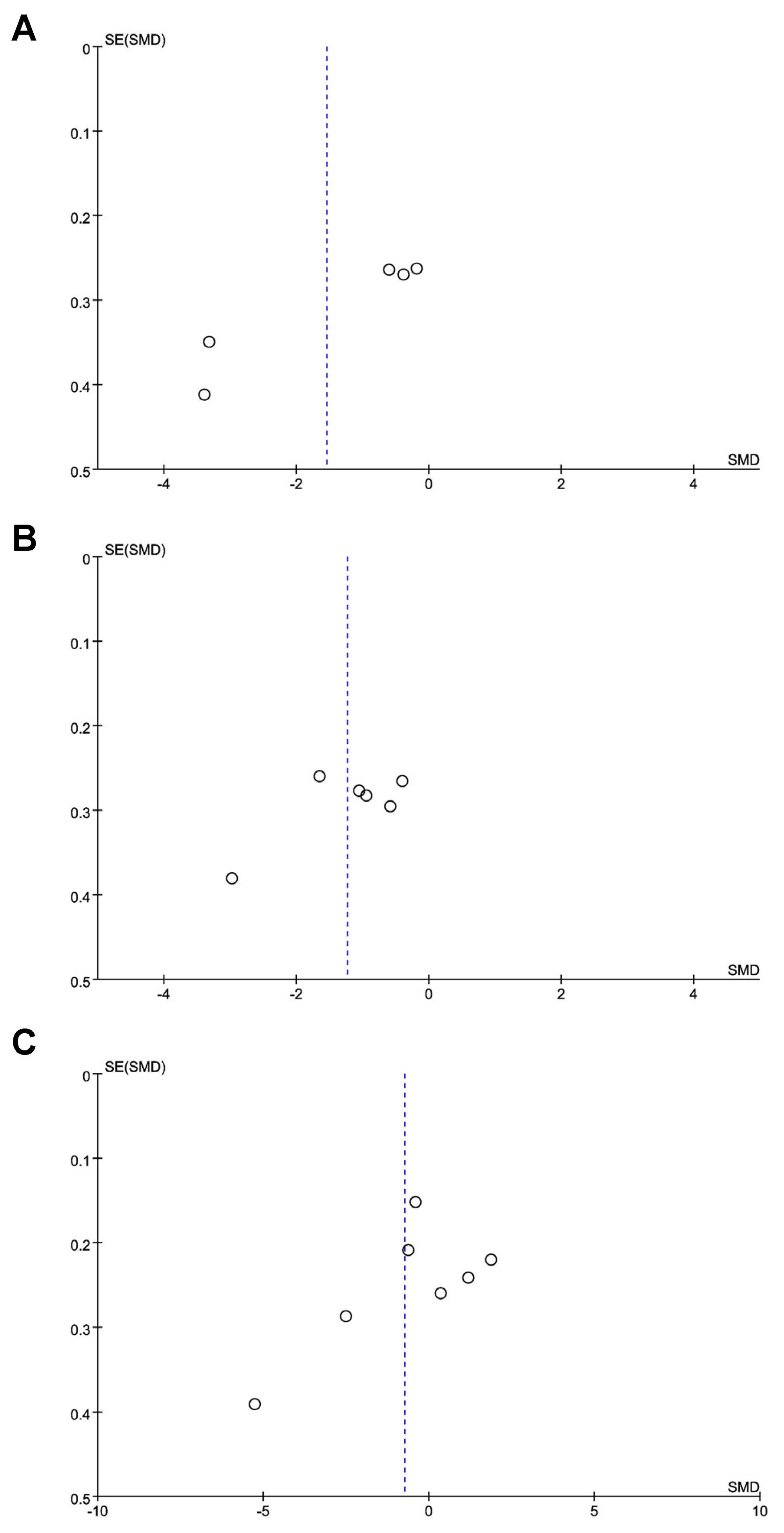

Supplement: Supplementary Figure 4 — The funnel plots were used to assess the publication bias for the STD Mean Difference of the DEX effects on the first flatus time (A), hospital stay (B) and postoperative extubation time (C). [file Image_4.jpeg]
